# Supplementary material for: Gellan Gum Promotes the Differentiation of Enterocytes from Human Induced Pluripotent Stem Cells
Source: Pharmaceutics. 2020 Oct 10;12(10):951. doi: 10.3390/pharmaceutics12100951 (PMC7599917; doi:10.3390/pharmaceutics12100951)
Supplement: Supplementary file 1 [file pharmaceutics-12-00951-s001.pdf]

pharmaceutics

Supplementary Materials: Gellan Gum Promotes the Differentiation of Enterocytes from Human Induced Pluripotent Stem Cells

Shimeng Qiu, Tomoki Kabeya, Isamu Ogawa, Shiho Anno, Hisato Hayashi, Tatsuro Kanaki, Tadahiro Hashita, Takahiro Iwao and Tamihide Matsunaga

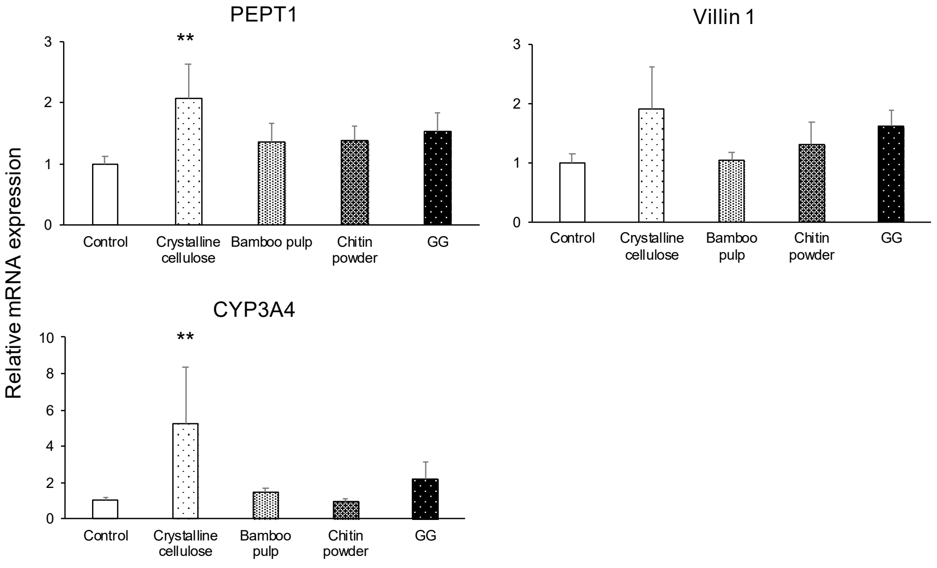

Figure S1. The comparisons of various kinds of fiber on enterocyte differentiation from human iPS cells. Relative gene expression levels of PEPT1, villin 1 and CYP3A4. All data are presented as mean ± S.D. (n = 3). Control = 1. Levels of statistical significance: $^{**}p < 0.01$.

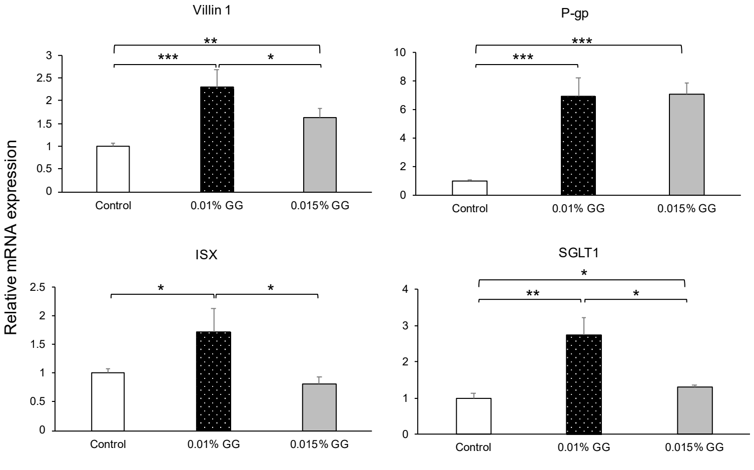

Figure S2. The effect of various GG concentrations on gene expression in iPS cell-derived enterocytes at the end of differentiation. Relative gene expression levels of villin 1, P-gp, ISX and SGLT1. All data are presented as mean ± S.D. (n = 3). Control = 1. Levels of statistical significance: $^{*}p < 0.05$, $^{**}p < 0.01$, $^{***}p < 0.001$.

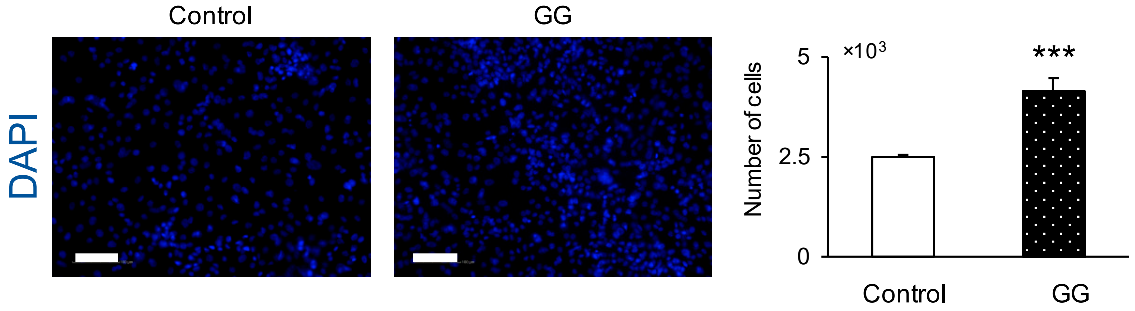

Figure S3. The effect of GG on the number of iPS cell-derived enterocytes at the end of differentiation. The number of differentiated enterocytes from human iPS cells, which belong to randomly selected fields of view, were counted. Scale bars = 100 μm. All data are presented as mean ± S.D. ($n = 3$). Control = 1. Levels of statistical significance: $^{***}p < 0.001$.

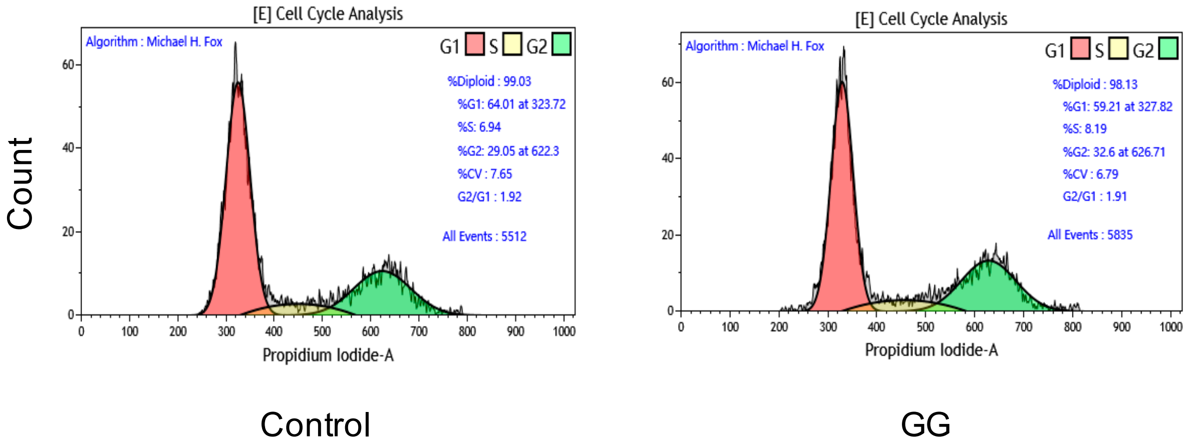

Figure S4. The effect of GG on the cell cycle analysis of iPS cell-derived enterocytes at the end of differentiation. PI staining of differentiated enterocytes.
